# Innovative Partnerships for Drug Discovery against Neglected Diseases

**DOI:** 10.1371/journal.pntd.0001221

**Published:** 2011-09-27

**Authors:** Palle H. Jakobsen, Ming-Wei Wang, Solomon Nwaka

**Affiliations:** 1 Novo Nordisk A/S, Bagsvaerd, Denmark; 2 The National Center for Drug Screening, Shanghai Institute of Materia Medica, Chinese Academy of Sciences, Shanghai, China; 3 UNDP/UNICEF/World Bank/WHO Special Programme for Research and Training in Tropical Diseases, World Health Organization, Geneva, Switzerland; McGill University, Canada

There is a compelling scarcity of pharmaceutical agents for efficacious, safe, and affordable treatment of neglected infectious or tropical diseases such as malaria, trypanosomiasis, leishmaniasis, dengue, lymphatic filariasis, and soil-transmitted helminths, despite their high prevalence in the developing world. Toxicity of drugs, microbial resistance patterns, and long courses of treatments are among the current challenges in effective management of neglected diseases.

Lack of commercial market is often outlined as the main factor for the limited number of drugs against neglected diseases. In the past 10 years, the product research and development (R&D) pipeline, including vaccines, diagnostics, and drugs, for neglected diseases has been supported through product development partnerships (PDPs) and others [1]–[3]. While R&D activities for vaccines and diagnostics for neglected diseases are centered on the identification and evaluation of biomarkers, development of new drugs is presently focused on small molecules with little or no ongoing effort for biopharmaceuticals.

Small molecule drug discovery and development is a lengthy, risky, and costly process. However, it has been shown that the cost of developing drugs for neglected diseases through public–private partnership (PPP) and network models may be significantly less, due to sharing the financial burden between various agencies, including government, philanthropic, and private agencies [3] and adopting rational criteria for lead progression [4].

Discovering leads with the potential to become therapeutics is a critical step in drug development. This involves a strong interplay between various disciplines and expertise in (i) biology, such as target identification and validation, assay development, screening, bioinformatics, and ADME/T (absorption, distribution, metabolism, and excretion/toxicology); and (ii) chemistry, including medicinal chemistry, compound libraries and knowledge management. Compound collections or libraries containing hundreds, thousands, or hundreds of thousands of small molecules or natural substances may be screened against molecular targets or whole cells with variable results. When active hits are identified from successful screens, they undergo extensive characterization and prioritization to rationalize further investment in optimizing them through extensive structure-activity relationship (SAR) analyses. These activities encounter harsh attrition along every step of the discovery value chain, not just at the level of a specific hit or lead, but also during the selection and validation of a molecular target as well as assay development. Because these lead discovery activities are of high risk, they tend not to receive much funding from the normal scientific granting bodies, and so there is less incentive for academia to work in this area [5].

In the absence of a perceived commercial market for drugs against neglected diseases, it is critical to devise sustainable approaches to stimulate and fund R&D activities in this field. In spite of the progress made by existing PDPs, there remain major hurdles to the discovery of new chemical entities (NCEs) against neglected diseases. An innovation gap has therefore been defined for a range of these diseases [4]. While some PDPs (e.g., Medicines for Malaria Venture and Drugs for Neglected Diseases Initiative) have done an excellent job in collaborating with industry and academic institutions to screen compound libraries against some of the disease pathogens in vitro, many have focused to a large extent on the so-called “low-hanging fruit”, i.e., reformulation or new combination of existing drugs or those developed for other indications (Table 1). To ensure a continued flow of promising candidates into late-stage development, discovery efforts must be maintained in a sustainable manner and with active participation of disease-endemic countries (DECs) [4]–[6].

**Table 1:** Product development partnerships (PDPs) focusing on drugs.

1) Institute of One World Health (http://www.iowh.org/) 2) Medicines for Malaria Venture (http://www.mmv.org/) 3) Drugs for Neglected Diseases Initiative (http://www.dndi.org/) 4) Global Alliance for TB Drug development (http://tballiance.org/)	
**Description**	○ Type of organization: not-for-profit ○ Operating model: virtual R&D ○ Approach: building partnership with pharmaceutical industry, biotechnology companies, and academic institutions; utilizing portfolio management approach ○ Funding: philanthropy, governments, etc. ○ Location: mainly in developed countries ○ Capacity building: limited

Several pre-competitive approaches have been suggested for overcoming these challenges, including a more coordinated collaboration mechanism involving multidisciplinary networks of investigators and partnerships between industry and public sector in both developed and developing countries [4], [5], [7]; a more open and cooperative model where information and knowledge are freely shared to support innovation [8], [9]; and the establishment of regional networks of R&D centers in developing countries to tackle the problems [10]–[13]. These more collaborative and empowering research environments would increase the efficiency and lower the cost of developing new, safe, and effective medicines, vaccines, and diagnostics while building indigenous innovation capacity in developing countries [4], [12]–[15]. It will also bring different stakeholders together to contribute their expertise and knowledge in a coordinated way [4], [5], [8]. For instance, industry can contribute its expertise, compound libraries, infrastructure, training, and monetary or other in-kind support; academic institutions can contribute basic research and understanding of pathogens, genomics, and whole cell assays; while governments and non-governmental organizations can contribute resources such as manpower and finance.

**Figure 1:**
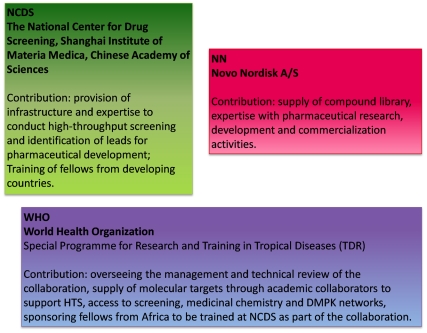
Examples of public–private partnerships in drug discovery against neglected diseases. **NCDS - The National Center for Drug Screening, Shanghai Institute of Materia Medica, Chinese Academy of Sciences.** Contribution: provides infrastructure and expertise to conduct high-throughput screening (HTS) and identification of leads for pharmaceutical development, as well as training of fellows from developing countries. **NN - Novo Nordisk A/S.** Contribution: supply of compound libraries, expertise with pharmaceutical research, development, and commercialization activities. **WHO - World Health Organization Special Programme for Research and Training in Tropical Diseases (WHO/TDR).** Contribution: oversees the management and technical review of the collaboration, supplies molecular targets through academic collaborators to support HTS, provides access to screening, medicinal chemistry, and DMPK networks, and sponsors fellows from Africa to be trained at NCDS as part of the collaboration.

A recurring difficulty for all screens against neglected diseases is the availability of high-quality compound libraries and the resources to support the prioritization and analysis of any resultant hits [5]. In Figure 1 we describe an innovative partnership that supports drug discovery against neglected diseases as well as training of African scientists through collaboration between a public institute (the National Center for Drug Screening, Shanghai [NCDS]), an international agency (the Special Programme for Research and Training in Tropical Diseases [WHO/TDR]), and a multinational biopharmaceutical company (Novo Nordisk [NN]).

**Figure 2:**
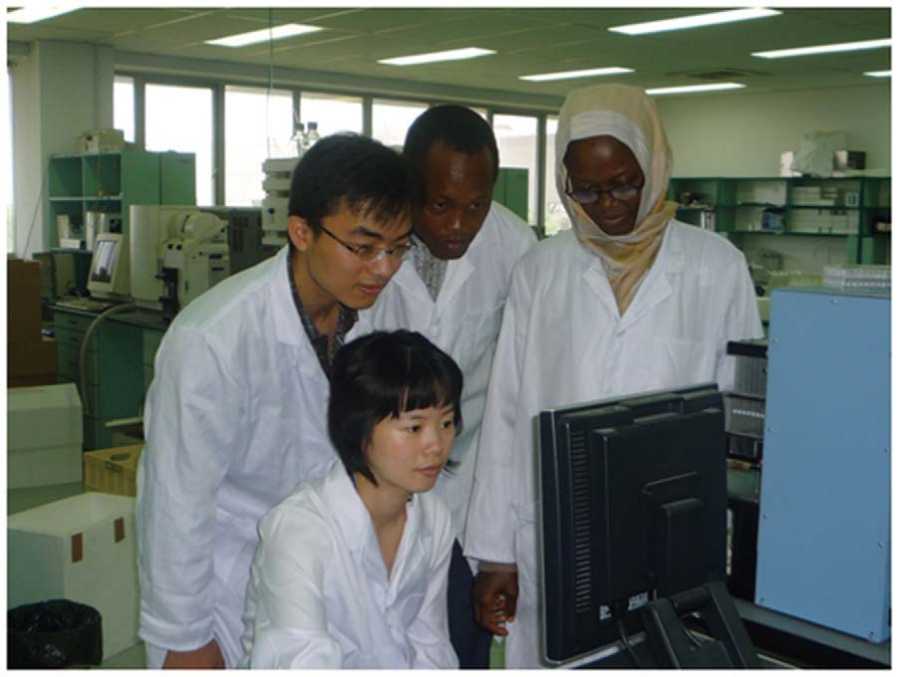
NCDS scientists and African fellows are analyzing the results obtained from a TDR commissioned HTS campaign against a molecular target in tuberculosis (July 2009).

This collaboration was launched in July 2009 following a contractual agreement of the parties and the initiation of the first high-throughput screening (HTS) campaign (Figure 2). The partnership focuses on hit-to-lead activities against a range of neglected diseases, including schistosomiasis and tuberculosis. It should be mentioned that some companies have established in-house research facilities to develop new drugs against neglected diseases. For example, Novartis has set up an institute in Singapore focusing on malaria, dengue, and tuberculosis, and AstraZeneca in India is working on tuberculosis. GSK in Spain is now operating as an open laboratory to support drug discovery for neglected diseases. While these are all excellent efforts, more efforts and investments are needed to support sustainable health innovation in developing countries.

Some recent inter-governmental actions have stressed the need to invest in building sustainable capacity for health innovation in the developing world. Of note is the Global Strategy and Plan of Action (GSPOA) on public health, innovation, and intellectual property, which aims to promote new thinking on innovation and access to medicines for diseases that disproportionately affect developing countries [10]. It also calls for stronger PPPs and North–South and South–South collaborations, as well as the establishment of regional and international networks in support of product innovation in disease-endemic regions. This initiative is best exemplified by the formation of the African Network for Drugs and Diagnostics Innovation (ANDI), which operates under a regional governance and management. ANDI hopes to provide a time-efficient, cost-effective, and inclusive model to meet critical health care challenges in the continent [11]–[13] (Table 2). It is anticipated that leads emerging from the NCDS, NN, and WHO/TDR collaboration could, for example, be further optimized and developed through regional innovation networks in developing regions like ANDI or other partners.

**Table 2:** Brief description of the African Network for Drugs and Diagnostics Innovation (ANDI).

○ The ANDI concept was launched in Abuja, Nigeria, in 2008 (http://www.andi-africa.org/) ○ ANDI is now hosted by the United Nations Economic Commission for Africa (UNECA) following the Memorandum of Understanding between the World Health Organization (WHO) and UNECA ○ ANDI has established a ministerial-level Board. The first Board meeting was held in January 2011 in Addis Ababa, Ethiopia. The Minister of Science and Technology of South Africa and Minister of Public Health and Sanitation of Kenya were elected as co-chairs of the ANDI Board	
**Vision**	Create a sustainable platform for R&D innovation in Africa to address Africa's own health needs
**Mission**	Promote and sustain African-led health product innovation that addresses African public health needs through the assembly of research networks, and build capacity to support human and economic development
**Progress to date and future activities**	○ 3 successful stakeholders meetings - first at Abuja in October 2008 in collaboration with the Nigerian government, second at Cape Town in October 2009 in collaboration with the South African government, and third at Nairobi in October 2010 in collaboration with the Kenyan government that attracted over 500 people in attendance ○ Development and endorsement of the strategic business plan for ANDI by stakeholders ○ ANDI Scientific and Technical Advisory Committee established ○ Review of the first Pan-African Centers of Excellence in health innovation completed ○ Review of the 1st ANDI call for projects in progress ○ Ongoing advocacy and resource mobilization ○ Establishment of 5 regional hubs in the 5 regions of Africa ○ Establishment and funding of ANDI portfolio of projects

A broader interest has been expressed by several stakeholder groups in Asia and some preliminary mapping of innovation centers is under way in China, India, and several Association of Southeast Asian Nations (ASEAN) countries. The Chinese NDI held its first meeting in October 2009 and is in communication with its neighboring counterparts with the goal of creating a pan-Asia network. The collaborative project, described in Table 2, as well as enthusiasm shown by other institutions in China, including the National Institute of Parasitic Diseases of the Chinese Center for Disease Control and Prevention and the Second Military Medical University, have made a significant contribution towards the establishment of the Chinese NDI (Table 3). Cooperation among African, Asian, and South American scientists is a good way to promote South–South collaboration and technology transfer.

**Table 3:** Brief description of the Chinese Network for Drugs and Diagnostics Innovation.

○ Founded in October 2009 with the support of WHO/TDR and China's CDC and Ministry of Science and Technology (http://www.asiandi.org/china/index.asp) ○ Participants include major universities, research institutes, hospitals, multinational pharmaceutical giants, and biotech companies	
**Mission**	○ Establishing a network that shares scientific information and technical resources to leverage product R&D activities nationwide ○ Developing workable mechanisms by which PPPs in neglected diseases can flourish in China ○ Assisting in intellectual property rights protection and advancing legitimate interests of the network members
**Activities**	○ R&D landscape mapping with initial phase of gap analyses on neglected disease in China completed. A formal report is being completed and will be publicly available in 2011.Organized an international workshop on quality in clinical research (June 2010).Networking with other regional networks, including representation of China in the third Stakeholders Meeting and High Level Forum of ANDI (October 2010, Nairobi, Kenya). ○ First and second annual meetings of the Chinese NDI held in October 2009 and January 2011, respectively.

In conclusion, we have described a few innovative public–private and North–South partnerships for drug development against neglected diseases. Such partnerships are highly relevant in supporting capacity building and establishment of R&D infrastructures in developing countries. The hope is that these and other emerging South–South activities will be scaled up and sustained to support the discovery, development, and delivery of new drugs, diagnostics, vaccines, and medical devices for diseases that disproportionately affect developing countries.
